# Intramedullary Kirschner wire fixation of displaced distal forearm fractures in children

**DOI:** 10.1186/s12891-023-06875-z

**Published:** 2023-09-21

**Authors:** Mohamed I. Abulsoud, Ahmed Saied Mohammed, Mohammed Elmarghany, Ahmed Elgeushy, Ehab Elzahed, Mohamed Moawad, Ehab A. Elshal, Mohamed F. Elhalawany, Yahia A. Hassanein, Amr A. Fouad, Ahmed R. Zakaria

**Affiliations:** 1 Department of Orthopedic Surgery, Faculty of Medicine, Al-Azhar University, Cairo, Egypt; 2 Department of Orthopedic Surgery, Helwan University, Helwan, Egypt

**Keywords:** Distal forearm fracture, Distal radius fractures, Intramedullary fixation, Kirschner wire, Pediatric wrist fractures

## Abstract

**Aim of the work:**

This study was designed to highlight internal fixation by intramedullary K-wires for displaced distal forearm fractures among children and analyze the results of this technique. We hypothesize that physis-sparing intramedullary fixation prevents displacement with a lower complication rate.

**Methods:**

This prospective case series involving 47 patients was conducted between February 2018 and December 2019. All patients with open physis presented with recent displaced distal forearm fractures were included, and all of them were treated with an intramedullary k-wire fixation for both bones with the assessment of the union rate, union time, suspected complication, radiographic evaluation, and functional outcome.

**Results:**

The study population consisted of 31 boys (66%) and 16 girls (34%). The mean age of the patients was 10.68 ± 2.728 years (range, 7–15 years). All fractures were united in a median of 6 weeks (range, 4–8 weeks), The functional outcome after 12 months was normal in 42 patients (89.4%), whereas, in five patients (10.6%), the functional parameters were minimally reduced. The median preoperative angulation improved from 36° (range, 24°–52°) preoperatively to 4° (range, 0°–10°) on immediate postoperative radiographs. After 12 months, the median angulation was 2° (range, 0°–7°) (*p* < 0.001). The angulation of the distal radius immediately after surgery and at the final follow-up was statistically correlated with the functional outcome (*p* < 0.001 and 0.002, respectively).

**Conclusion:**

This technique provides a good result with less susceptibility to re-displacement and low complication rates.

**Level of evidence:**

Level IV.

## Introduction

Fractures of the distal radius and forearm are among the most common injuries in children, boys are more affected than girls especially the older age group around 12 years, while younger age groups with complete fractures may require surgical treatment more often [1]. Radius fractures account for 20% of all childhood fractures, and 62% of them are radial metaphyseal fractures [2].

The method of treatment is based on fracture type: un-displaced fractures can be treated with a splint or cast; however, displaced fractures are treated with either manipulation and immobilization with a cast under anaesthesia or manipulation and internal fixation [3].

The rate of re-displacement after manipulation of displaced distal radius fractures is approximately 21%–39%; many predisposing factors for re-displacement have been reported, most importantly, associated ulnar fracture, the degree of initial displacement, and casting and padding techniques [4–8].

Although the capability of such fractures to remodel with good functional outcomes, A re-displaced fracture is one of the most unpleasant scenarios for treating physicians as it may require further corrective surgery or be left to heal with either remodelling or mal-unite with poor functional outcomes. Therefore, percutaneous Kirschner (K) wire fixation has been recommended to avoid re-displacement; however, it comes with the possibility of complications [9]. Meanwhile, some authors [7] have advocated performing primary K-wire fixation for all cases of displaced distal radius fractures even when a satisfactory closed reduction has been achieved. Others, including Proctor et al. [10], have advocated performing fixation in all cases when a perfect reduction cannot be achieved, whereas Prevot et al. [11] have recommended fixation for instability and irreducibility.

The rate of complications following surgical treatment for distal radius fractures in children ranges from 6 to 80%, The most common complications following K-wire fixation are loss of reduction, pin tract infections, sunken K-wires, neuropraxia of the superficial radial nerve, and tendon irritation [8, 12]. The wide range in the literature is not the same as in adults where dates are similar in different reports in different times, sample sizes, and places giving more reliable reference and robust evidence for the real rate of complications [13–15].

This study aims to evaluate the radiographic and functional outcomes after intramedullary K-wire fixation of distal forearm fractures in children with an explanation of the surgical technique, and the rate of suspected complications.

## Methods

### Study design and participants

This is a prospective study that included 47 skeletally immature consecutive.

Ethical approval was obtained from the Institutional Ethical Committee and conducted in Al-Azhar University Hospitals, Cairo, Egypt from February 2018 to December 2019. All procedures performed in this study were in accordance with the ethical standards of the institutional research committee and with the 1964 Helsinki Declaration and its later amendments or comparable ethical standards. Informed consent was obtained from all individual participants’ guardians who were included in the study according to the rules of the hospital research ethical committee.

The Strengthening the Reporting of Observational Studies in Epidemiology (STROBE) guidelines for cohort studies were followed.

### Inclusion and exclusion criteria

Patients with a recent, displaced distal forearm fracture (metaphyseal distal radius fracture associated with distal ulnar fracture) treated with internal fixation by intramedullary K-wire for both the radius and ulna were included in the study, We can define skeletally immature children as those who have open radial and ulnar physis on plain radiographs, The initial displacement has been evaluated and graded according to Mani et al. [16]; cases with grade 3 or 4 displacements were included.

Patients with other types of forearm fractures, such as non-displaced distal forearm fractures, Galeazzi fractures, diaphyseal fractures, Physeal fractures, pathological fractures, associated upper limb fractures, and open fractures, and those more than 16 years old were excluded from this study.

### Surgical technique

All operations were performed according to the technique described under general anaesthesia. The patient’s position was supine. A pneumatic arm tourniquet was typically used for hemostasis.

**Fig. 1:**
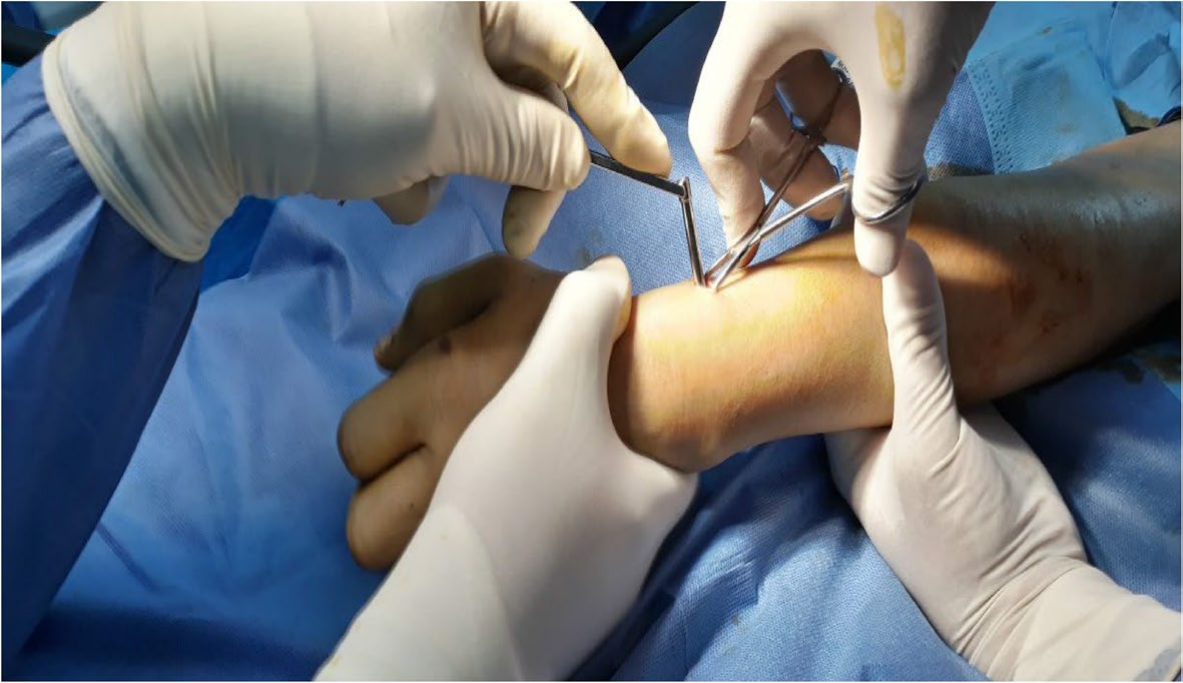
The first radial skin incision with the insertion of a sleeve before the insertion of the drill bit

**Fig. 2:**
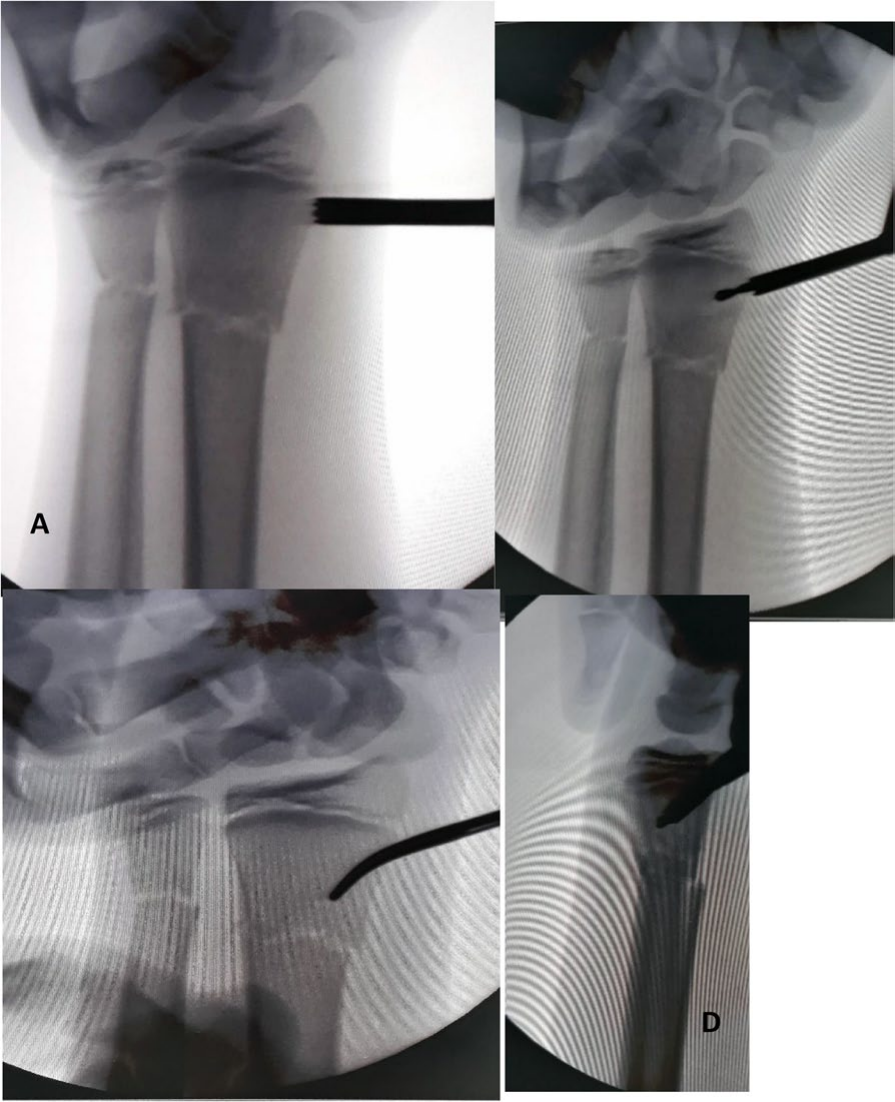
**A** Fluoroscopic image showing sleeve insertion on the radius. **B**, **C** Fluoroscopic images showing the insertion of the drill bit and checking its position on anteroposterior and lateral projections. **D** Re-establishing the track for the K-wire using a hemostat

A 2-cm skin incision was made at the level of the distal radius, proximal to the physis with dissection of soft tissue protecting the superficial radial nerve and extensor tendons of the first and second compartments using small Langenbeck’s retractors. When the bone was reached, a 2.5-mm drill bit was used to open the medullary canal under fluoroscopic guidance using a sleeve to protect the soft tissues (Figs. 1 and 2).

**Fig. 3:**
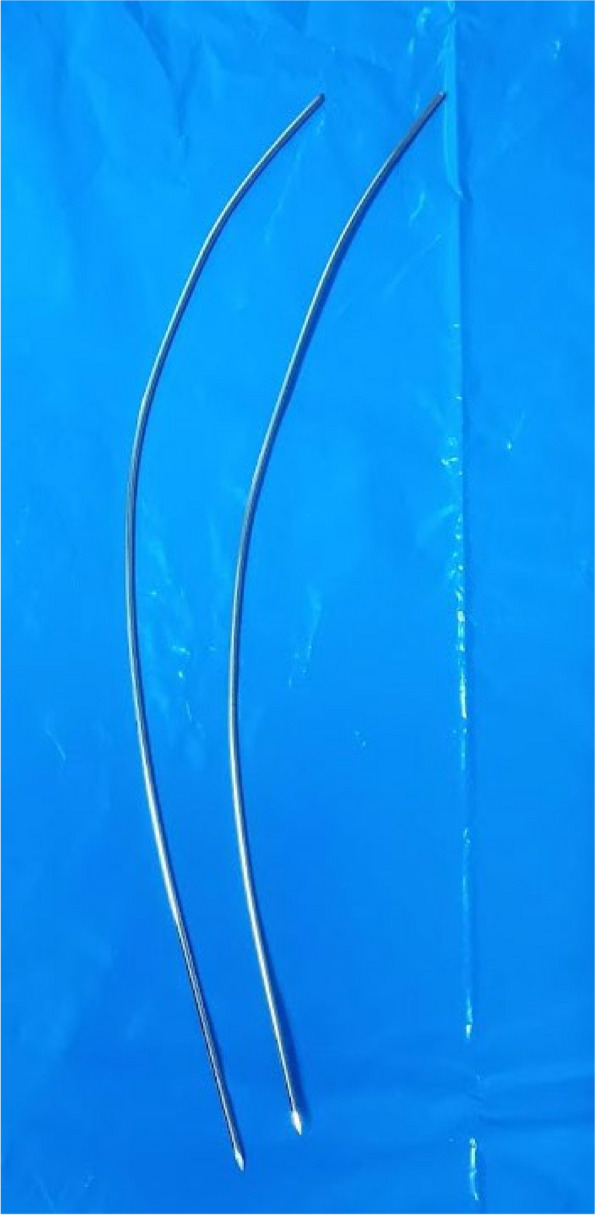
The pre-bent blunt-tipped K-wires were used for the fixation of distal radius fractures

Then, a hemostat was used to make the direction for the K-wire; during this time, the preliminary reduction was performed. Then, a 1.6–1.8 pre-bent K-wire was passed after cutting its trocar tip (Fig. 3), which was left long to occupy the entire length of the radius.

**Fig. 4:**
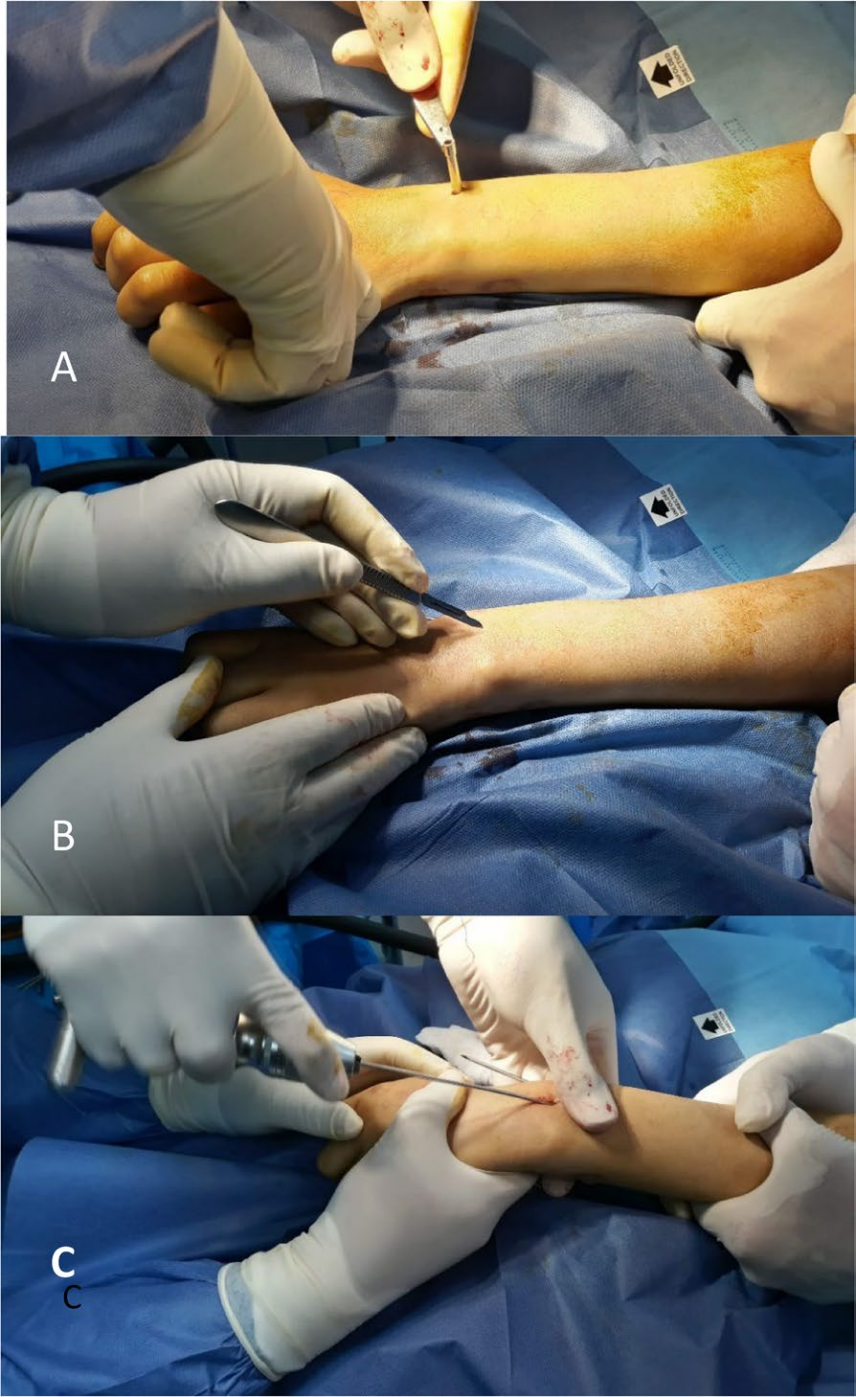
The steps of the insertion of the second K-wire in the dorsal side of the radius. **A** Skin incision. **B** Insertion of the sleeve for insertion of the drill bit. **C** Insertion of the K-wire by a manual handle

**Fig. 5:**
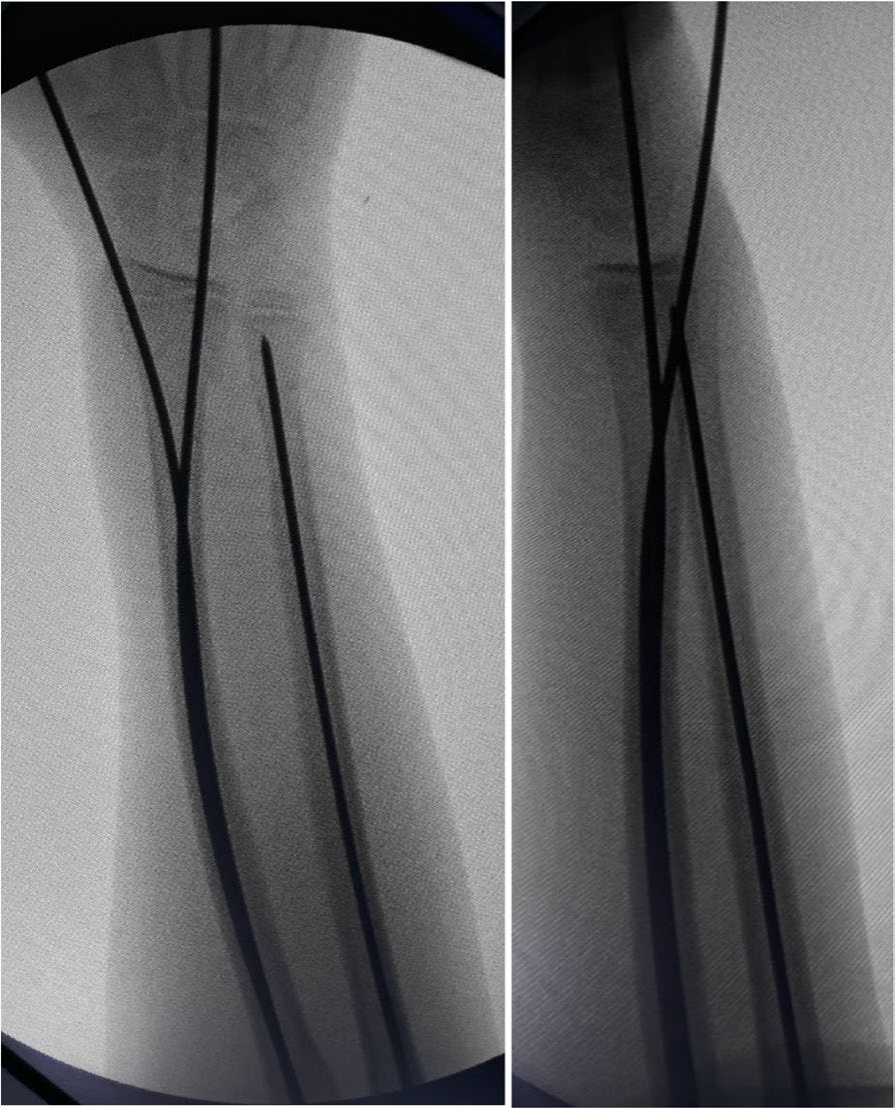
Fluoroscopic image shows the final check after insertion of the intramedullary k wires showing good reduction and stable fixation

**Fig. 6:**
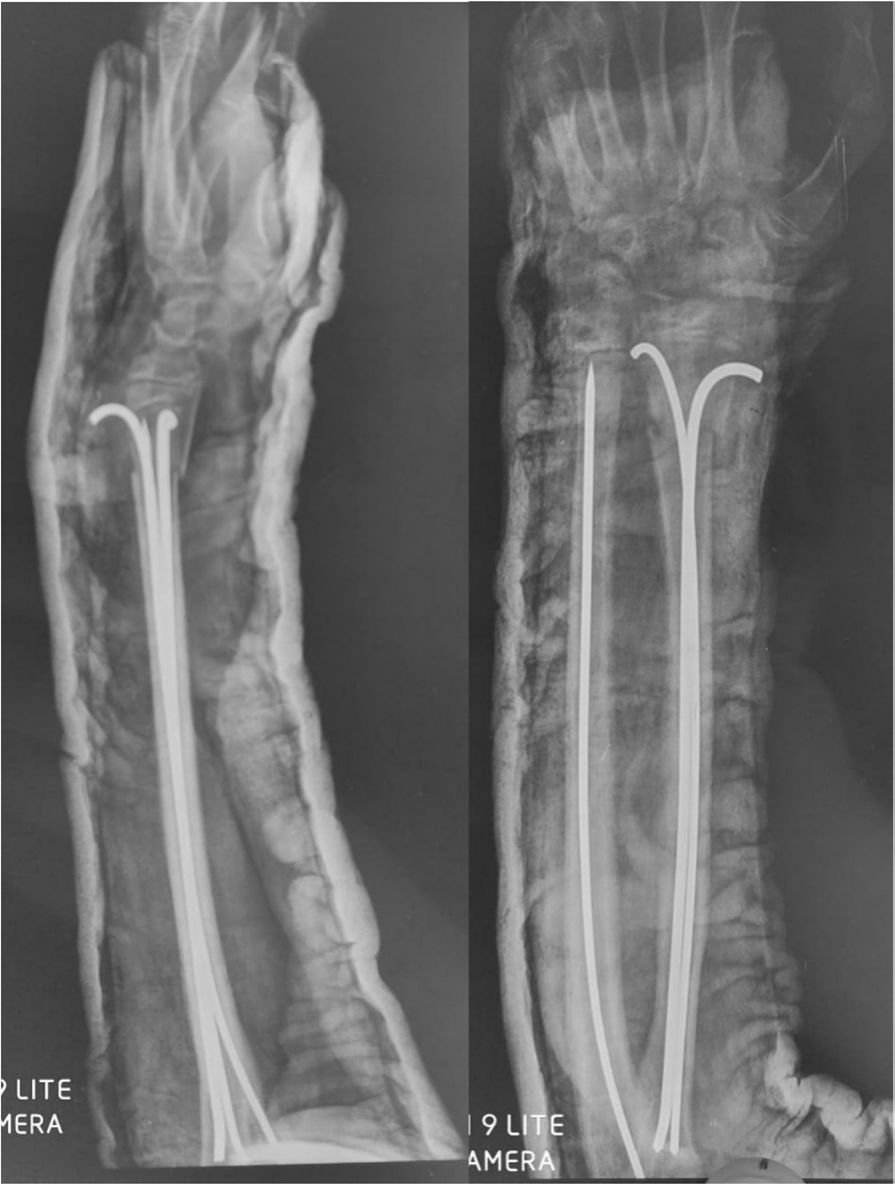
Anteroposterior and lateral X-ray views showing the immediate postoperative radiograph of the case illustrating the surgical technique

A second wire was inserted from the ulnar side between the 4th and 5th extensor compartments using the same technique (Fig. 4). Then, the ulna was fixed using an intramedullary K-wire reduction from the olecranon, which runs distally. The skin was closed by simple stitches (Fig. 5). After the final check of reduction and fixation, a sugar-tong splint was applied for 3–4 weeks (Fig. 6).

**Fig. 7:**
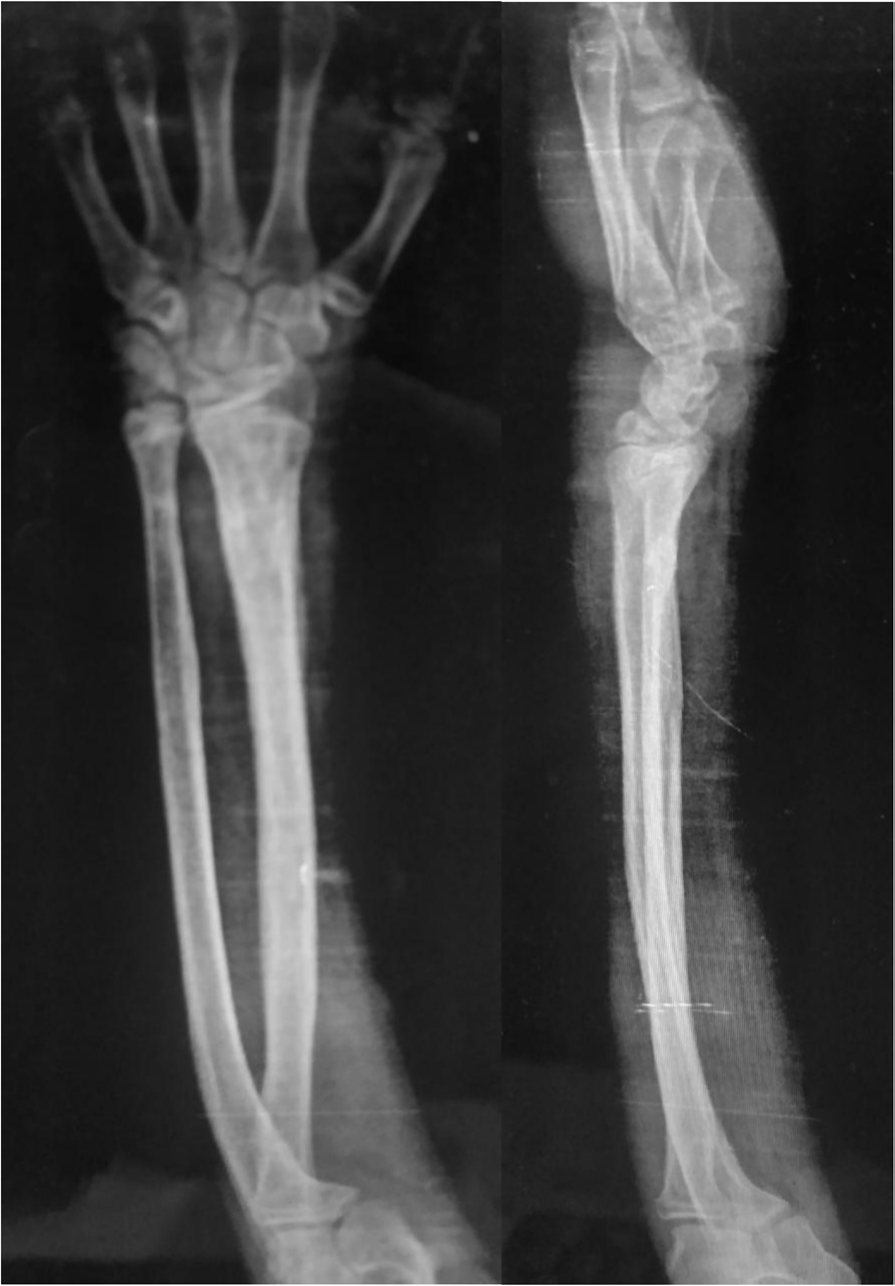
Anteroposterior and lateral X-ray views show the final follow-up 1 year postoperatively

The patients were encouraged to visit the outpatient clinic every 2 weeks. Serial radiographs were taken to check for healing and re-displacement. When the union was achieved, the patients were scheduled for K-wire removal under general anaesthesia (Fig. 7).

### Outcome measurement

The collected data from the X-ray scans were analyzed as follows. The preoperative and postoperative angulations of the fractures were measured in the lateral plane. Additionally, the rate of union, rate of re-displacement, remodelling capacity during follow-up visits, and complications (e.g., infections, re-displacement, nonunion, refractures, and nerve damage), if present, were recorded. Postoperatively, the functional and radiological outcomes were evaluated after 12 months: the functional outcomes at follow-up were classified into normal, mildly reduced, moderately reduced, or severely reduced. The mildly reduced function was defined as less than 10° restriction in forearm rotation compared with the other side with spontaneous resolution. The moderately reduced function was defined as more than 10° restriction in forearm rotation compared with the other side, which was resolved by physiotherapy. Meanwhile, the severely reduced function was defined as more than 30° restriction, which cannot be resolved by physiotherapy and requires further management. Radiographic outcomes were determined by measuring the angulation on lateral forearm radiographs and the existence of growth arrest or translation on anteroposterior radiographs.

**Fig. 8:**
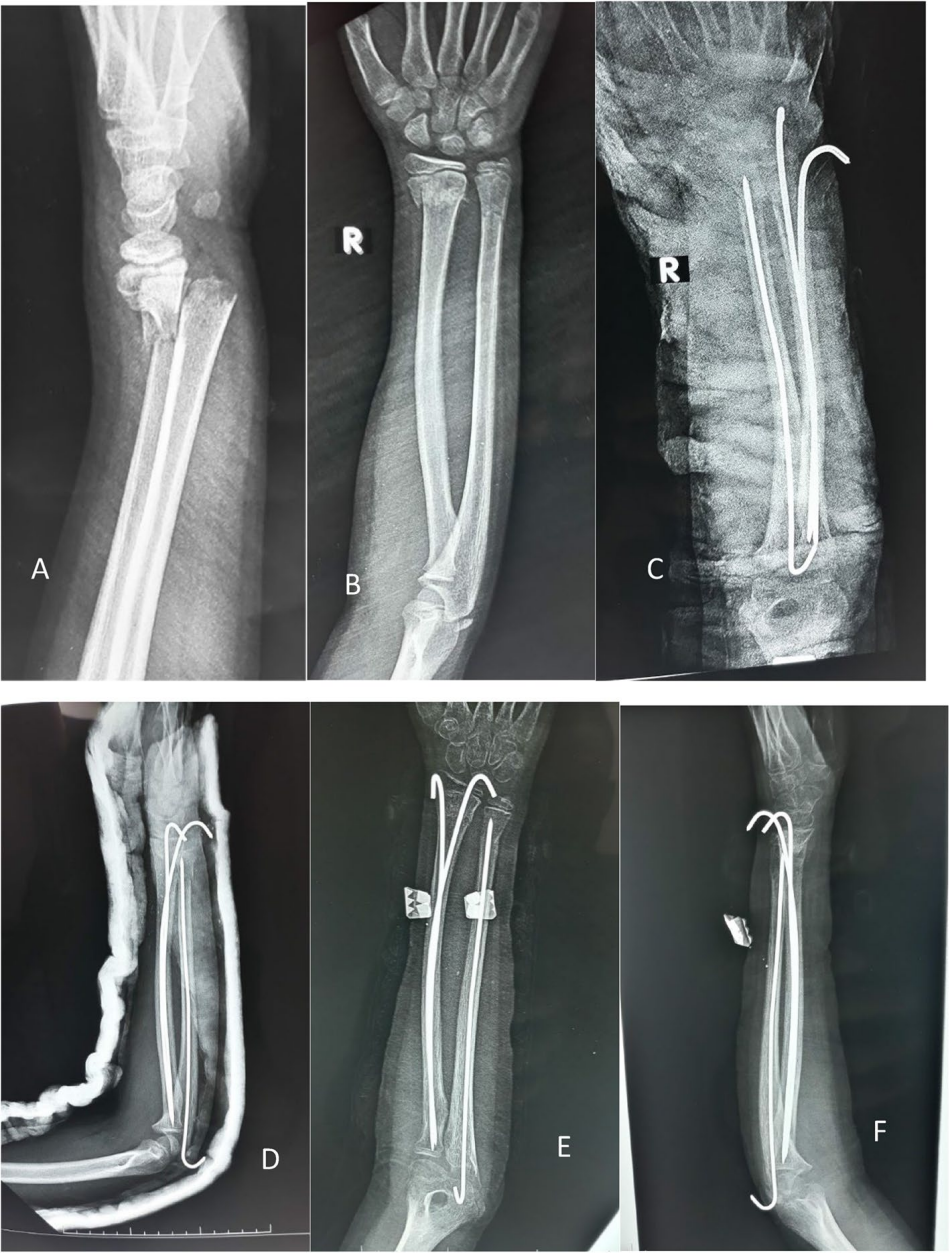
**A** and **B** x-rays show Anteroposterior and lateral x-rays show a fracture distal forearm in a female 9-year-old patient. **C** and **D** postoperative X-rays. **E** and **F** x-rays after 6 weeks show stable fixation with a solid union

**Fig. 9:**
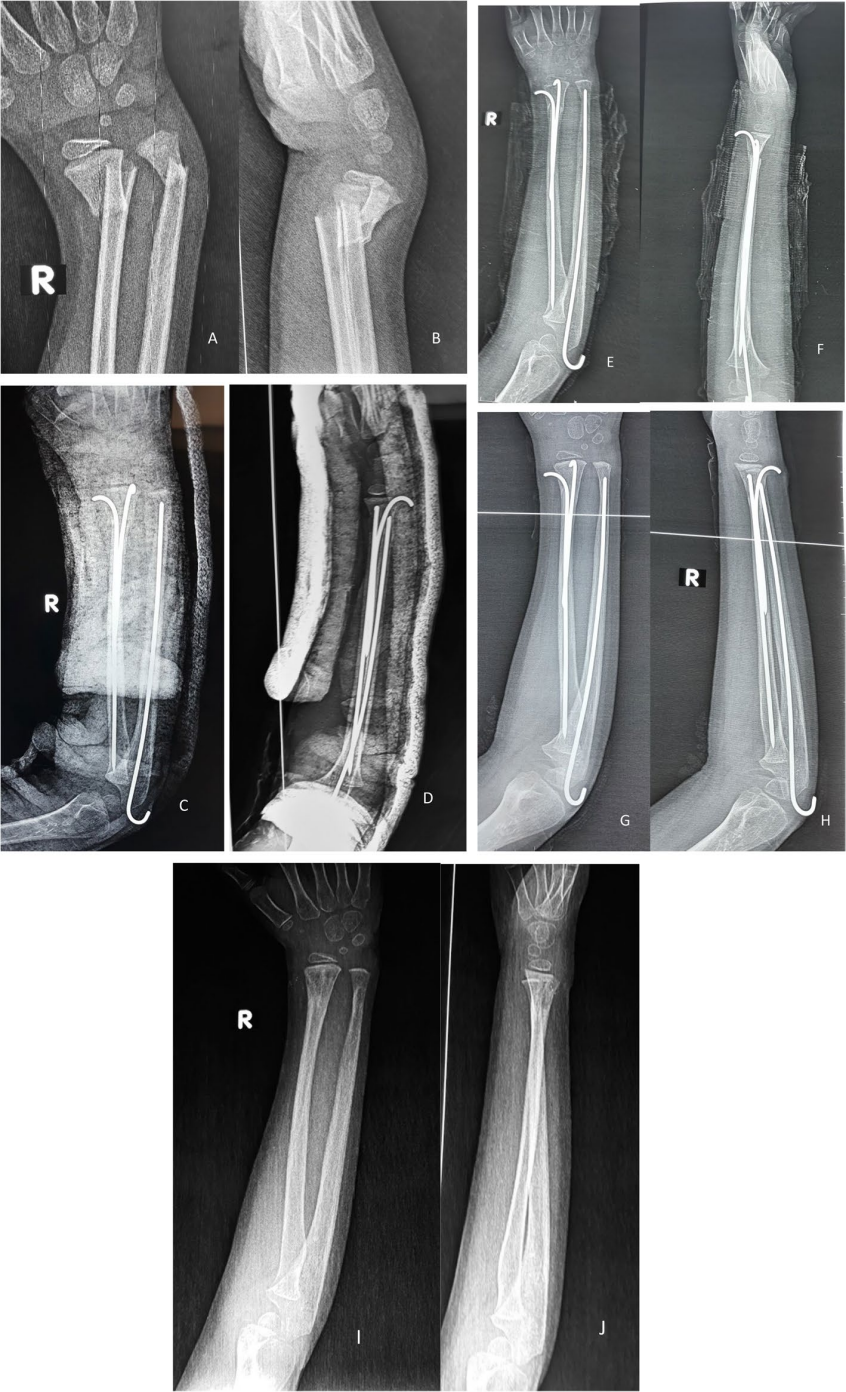
**A** and **B**. Anteroposterior and lateral X-rays show a distal forearm fracture in a male 7-year-old patient. **C** and **D**. Anteroposterior and lateral X-rays show the immediate postoperative radiograph. **E** and **F**. Anteroposterior and lateral X-rays show the follow-up radiographs 4 weeks postoperatively. **G** and **H**. Anteroposterior and lateral X-rays show the follow-up radiographs 8 weeks postoperatively with a complete union of both fractures. **I** and **J**. Anteroposterior and lateral X-rays show the follow-up radiographs at the end of follow-up after K-wire removal

Figures 8 and 9 present the cases.

### Data analysis

Data were analyzed using the Statistical Package for the Social Sciences (version 25, Armonk, NY, USA). The data were presented as mean and range for quantitative variables, whereas, for qualitative variables, data were presented as frequency and percentage.

The association between qualitative variables was assessed using the chi-square test or the nonparametric Fisher’s exact test, as appropriate. The Friedman and Wilcoxon signed-rank tests were used to compare variables before and after surgery. *P*-values of less than 0.05 were used to denote statistical significance. The relative percentage change was calculated as follows:

$$\mathrm{Relative}\;\%\;\mathrm{change}=\left[\left(\mathrm{after}\;\mathrm{activation}-\mathrm{before}\;\mathrm{activation}\right)/\mathrm{before}\;\mathrm{activation}\right]\times100$$

## Results

The study included forty-seven patients who were presented with distal forearm fractures (metaphyseal distal radius fracture associated with distal ulnar fracture). The mean age of the patients was 10.68 ± 2.728 years (range, 7–15 years). Among the 47 patients, 31 were boys (66%) and 16 were girls (34%). The median time of surgery was 3 days (range, 1–9 days) (Table 1).

**Table 1:** Clinical characteristics of the patients

Parameter	Data
Age (years)	
Mean ± SD (range)	10.68 ± 2.728
	(7–15)
Gender	
Male	31 (66%)
Female	16 (34%)
Duration (days)	
Median (range)	3 (1–9)
Time to union (weeks)	
Median (range)	6 (4–8)
Removal time (weeks)	
Median (range)	8 (5–10)
Primary displacement	
Grade III	17 (36.2%)
Grade IV	30 (63.8%)
Displacement postoperative	
Grade I	47 (100%)
Outcome	
Normal	42 (89.4%)
Minimally reduced	5 (10.6%)

All fractures were united in a median period of 6 weeks (range, 4–8 weeks). The median time from the insertion to the removal of K-wires was 8 weeks (range, 5–10 weeks). Preoperatively, the displacement of the fractures was grade 3 according to the Mani grading system in 17 patients (36.2%), whereas, in 30 patients (63.8%), the displacement of the fracture was grade 4 (Table 1).

The functional outcomes after 12 months were normal in 42 patients (89.4%), whereas, in five patients (10.6%), functional parameters were minimally reduced.

**Fig. 10:**
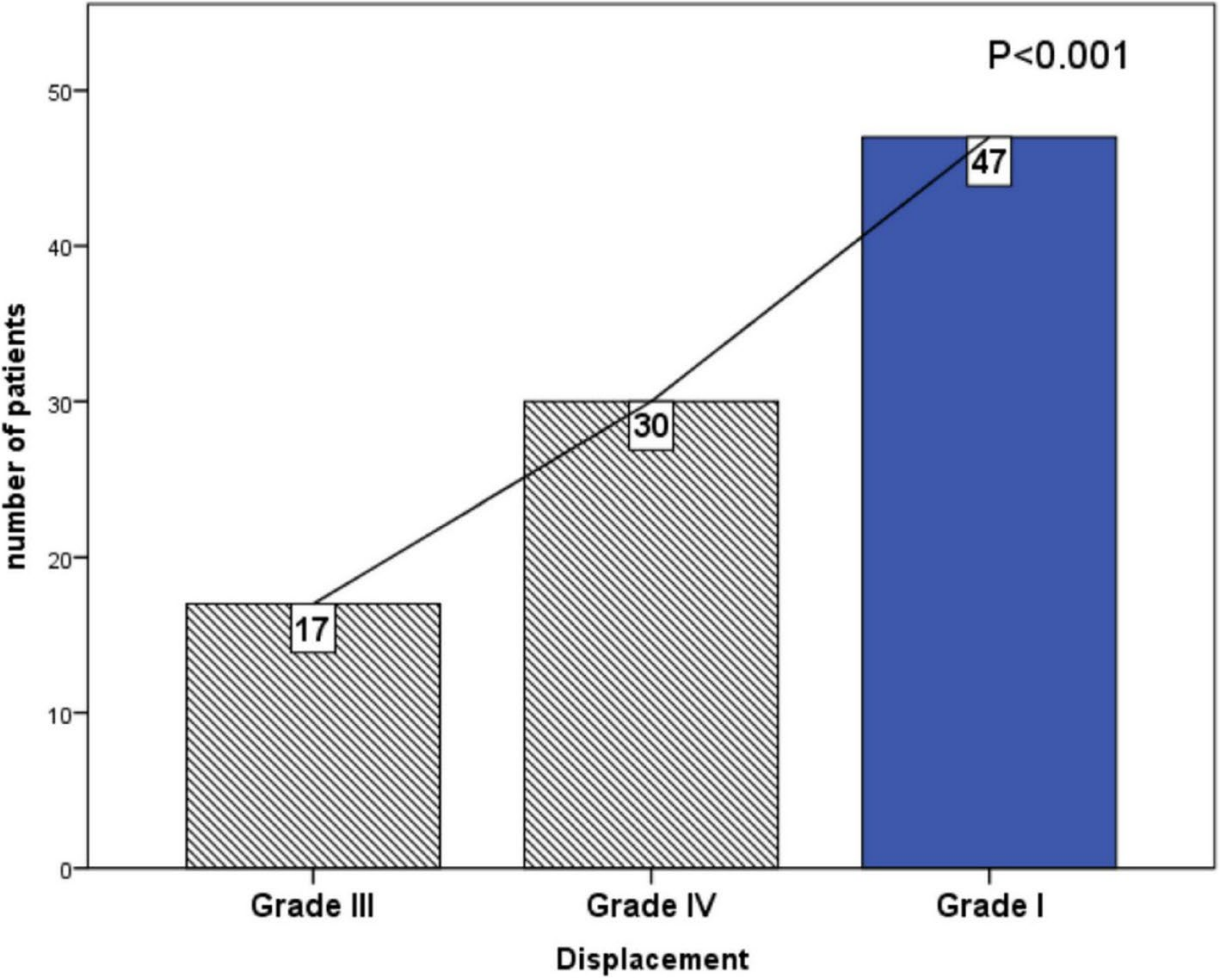
A chart shows the significant differences between preoperative displacement (grey shaded boxes) and postoperative displacement (blue box)

Subgroup analysis of degrees of angulation of the distal radius on lateral radiographs showed a statistically significant difference between all groups (*p* < 0.001) (Fig. 10 and Table 2).

**Table 2:** Comparison between preoperative angulation, immediate postoperative angulation, and angulation at the final follow-up after 12 months

Variable	Angulation preoperative	Angulation immediately postoperative	Angulation final	*P*-value
Median (range)	36 (24–52) ^a^	4 (0–10) ^b^	2 (0–7) ^c^	< 0.001

Variables with different letters are statistically significant; *p* < 0.005

Regarding the radiographic measurement of the angulation of the distal radius fractures on lateral views, the median preoperative angulation was 36° (range, 24°–52°), which improved to 4° (range, 0°–10°) on immediate postoperative radiographs, with a decrease in percentage change by 88.9%. After 12 months, the median angulation was 2° (range, 0°–7°), with a decrease in percentage change by − 50% compared with that on immediate postoperative radiographs and by − 94.4% compared with preoperative angulation (*p* < 0.001 for all groups) (Table 3).

**Table 3:** Comparison between angulations (pre- and postoperative)

Assessed variable		Percentage change	Z	*P*-value*
**Angulation preoperative**	**Angulation immediately postoperative**	− 88.9%	− 5.973	< 0.001
	**Angulation final**	− 94.4%	− 5.970	< 0.001
**Angulation immediately postoperative**	**Angulation final**	− 50%	− 4.994	< 0.001

^*^The Wilcoxon signed-rank test

### Association of patients’ outcomes with the assessed clinical features

Regarding the association of the functional outcome with the collected variables, it was found that the immediate postoperative angulation of the distal radius and the angulation at the final follow-up were statistically significant (*p* < 0.001 and 0.002, respectively) (Fig. 2). Meanwhile, the primary displacement, age, gender, preoperative duration, union time, and removal time were not statistically significant, indicating that the quality of reduction is a potential predictor for restoration of the normal range of motion (Table 4).

**Table 4:** Association between patients’ outcomes and the assessed clinical features

	**Outcome**		***P*-value**
	**Normal**	**Minimally reduced**	
Age (years)	9 (5–15)	10 (8–11)	0.627
Gender			
Male	28 (66.7%)	3 (60.0%)	0.766
Female	14 (33.3%)	2 (40.0%)	
Duration (days)	3 (1–9)	5 (2–8)	0.207
Time to union (weeks)	6 (4–8)	7 (5–8)	0.445
Removal time (weeks)	8 (5–10)	8 (6–10)	0.294
Angulation preoperative	36.5 (24–52)	36 (29–39)	0.717
Angulation immediately postoperative	3 (0–9)	9 (8–10)	**< 0.001**
Final angulation	2 (0–7)	5 (4–5)	**0.002**
Primary displacement			
Grade III	15 (35.7%)	2 (40.0%)	1
Grade IV	27 (64.3%)	3 (60.0%)	

Regarding complications, one patient (2.1%) had a superficial infection at the radial K-wire sites where all of them spontaneously improved with local wound care and oral antibiotics for 3 days—no case of nonunion, refractures, or pin migration at the final follow-up.

## Discussion

This study was designed to describe and evaluate the results of intramedullary K-wire fixation of displaced unstable distal forearm fractures in children and adolescents with open physis as an option to overcome the complications of closed reduction only and cross-pinning as the available options for treatment.

The rate of secondary displacement in displaced distal forearm fractures is high, according to the study by van Egmond et al. [17]. The rate of significant secondary displacement after closed reduction under general anaesthesia was 43.7%. The associated distal ulnar fracture with displaced distal radius fracture is a significant risk factor for re-displacement after closed reduction and immobilization along with initial displacement and the quality of reduction [18–20]. Based on this evidence, performing primary internal fixation for such fractures is recommended to avoid complications and the hazards of multiple unnecessary anaesthesia exposures.

The cross-pinning of metaphyseal distal radius fractures has been described by several authors and is the standard of care for unstable fractures [3, 7, 21]; however, cross-pinning has the disadvantage of a relatively high rate of complications. In a systematic review by Khandekar et al. [22], the rate of pin site infection was as high as 52%. Buried K-wire has been reported in 30% of cases and neurapraxia in 9% of cases, according to the study by Ramoutar et al., which involved 248 patients [23]. Secondary displacement requiring revision has been reported in 2% of cases, K-wire migration in 6% of cases either inward or outward, and pin tract infection in 3% of cases. In an earlier study by Miller et al. [24], which involved 34 patients, 16 patients underwent cross-pinning, two (12.5%) of whom had pin tract infection and two (12.5%) of whom presented with pin migration. In the study by Luscombe et al. [25], three of seven patients who underwent cross-pinning fixation needed re-manipulation (42.9%); however, the small sample size does not support such a high rate of complications in practice.

Another disadvantage of cross-pinning of the distal radius metaphysis is the difficulty in engaging the opposite cortex, requiring several trials, which increases the risk of fracture of the distal fragment and physeal growth arrest [26].

Several options have been utilized for the management of pediatric distal radius fractures, Varga et al., [27] described the use of short double intramedullary elastic stable nails for fixation of distal radial fractures, the study included 24 patients with an average age of 9.8 years old, the union was achieved in all cases with a full range of motion however, 3 cases (12.5%) suffered from skin irritation because of the presence of the edge of the elastic nail.

The Epibloc system has been used to fix such fractures, in comparison with crossed pinning, the use of the Epibloc system led to better functional recovery, and better restoration of the range of motion, with less need for physiotherapy [28, 29].

Plate fixation was also described, in the study of Van Egmond et al., [30] Plate fixation was used in 26 patients with a median age of 12.5 years (IQR: 9–15), and the grip strength and range of motion were similar to non-injured side, however, the plate was required to be removed in 58% of cases, the invasiveness of plate fixation and plate removal plus the total cost and surgery time makes such option could resort either in neglected cases and keeps k-wire fixation first line of treatment in the management of displaced simple recent fractures in all pediatric age groups [31].

The results of this study are encouraging, especially since the technique is easy and not time-consuming and preserves the physis with minimal complications. Postoperatively, all patients had no translation, and the angulation significantly improved postoperatively compared with that measured preoperatively. The final follow-up showed statistically significant improvement in angulation compared with those measured immediately after surgery; this could be explained by the remodelling capacity of the bone in patients with open physis. The range of motion was restored in 89.4% of the patients, whereas only 10.6% of the patients had minimal restrictions on the range of motion, which resolved spontaneously.

Subgroup analysis has shown that dorsal postoperative angulation correlates significantly with the functional outcome, indicating that care should be devoted to the quality of reduction intraoperatively. Residual angulation and rotation significantly affect the range of motion, and any angulation of more than 10° should be corrected to avoid motion restrictions and late midcarpal instability of the wrist [32].

Using a splint for 3–4 weeks is recommended to minimize the risk of displacement of K-wire migration until the early signs of union appear on follow-up radiographs. Such a short period in children did not lead to stiffness of either the wrist or elbow. The K-wire should be buried to reduce the incidence of pin tract infection. Such a point may illustrate the difference between the results of this technique and those of traditional cross-pinning.

One of the obstacles encountered in the study design is the lack of a universally agreed functional outcome for pediatric wrist fractures. In the literature, several clinical and radiographic methods were found, and the reported outcomes were heterogeneous [33]. The traditional scores used in adults, such as the Modified Mayo Wrist Score and Disabilities of the Arm, Shoulder, and Hand score, cannot be applied to children, especially in the younger age group. Therefore, the outcome measure that was used by Ramoutar et al. [23] has been used in this study as it is a simple, effective, and reliable method, allowing for a more comprehensive measurement of functional outcomes in pediatric patients with wrist fractures.

The standard wrist parameters of adults on plain X-rays (i.e., radial height, radial inclination, and radiocarpal angle) are variable according to age and gender, especially when used in children less than 12 years old [34]. The use of such parameters in assessing the quality of reduction will be sophisticated and may be misleading as they are correlated with the chronological (or even bone) age.

Although this study described a novel technique that preserves physics and leads to excellent functional outcomes, it has some limitations. The study lacks a control group and depends on the results from a single centre. A randomized control multi-centre trial is needed to compare the efficacy of the intramedullary fixation technique with that of standard cross-pinning in displaced unstable distal forearm fractures. The sample size is relatively small, however, the study included patients with different age groups of children and adolescents, and the data extracted was sufficient for statistical work, another limitation is that the technique needs a secondary procedure for hardware removal.

In conclusion, physis-sparing intramedullary fixation using K-wires for distal forearm fractures in skeletally immature populations is a safe and effective treatment option and is associated with stable fixation without loss of reduction and minimal complications.

## Data Availability

The datasets used and analyzed during the current study are available from the corresponding author upon request.

## References

[1] Bergkvist A, Lundqvist E, Pantzar-Castilla E (2023). Distal radius fractures in children aged 5–12 years: a Swedish nationwide register-based study of 25 777 patients. BMC Musculoskelet Disord.

[2] Azad A, Kang HP, Alluri RK, Vakhshori V, Kay HF, Ghiassi A (2019). Epidemiological and treatment trends of distal radius fractures across multiple age groups. J Wrist Surg.

[3] Jordan RW, Westacott DJ (2012). Displaced paediatric distal radius fractures–when should we use percutaneous wires?. Injury.

[4] Gibbons CL, Woods DA, Pailthorpe C, Carr AJ, Worlock P (1994). The management of isolated distal radius fractures in children. J Pediatr Orthop.

[5] Schneider J, Staubli G, Kubat S, Altermatt S (2007). Treating displaced distal forearm fractures in children. European J Trauma Emerg Surg.

[6] Voto SJ, Weiner DS, Leighley B (1990). Redisplacement after closed reduction of forearm fractures in children. J Pediatr Orthop.

[7] Zamzam MM, Khoshhal KI (2005). Displaced fracture of the distal radius in children: factors responsible for redisplacement after closed reduction. The J Bone Joint Surg.

[8] Wasiak M, Piekut M, Ratajczak K, Waśko M (2023). Early complications of percutaneous K-wire fixation in pediatric distal radius fractures-a prospective cohort study. Arch Orthop Trauma Surg.

[9] Turner RG, Faber KJ, Athwal GS (2007). Complications of distal radius fractures. Orthop Clin North Am.

[10] Proctor MT, Moore DJ, Paterson JM (1993). Redisplacement after manipulation of distal radial fractures in children. J Bone Joint Surg.

[11] - Prévot, N., Salanne, P., Longis, B., Surzur, P., & Mouliès, D. (1997). Place du traitement orthopédique dans les fractures du quart inférieur des deux os de l'avant-bras chez l'enfant. A propos de 152 cas [Value of orthopedic treatment of distal fractures of the forearm in children. Apropos of 152 cases]. Revue de chirurgie orthopedique et reparatrice de l'appareil moteur, 83(3), 251–258. 9255361

[12] Subramanian P, Kantharuban S, Shilston S, Pearce OJ (2012). Complications of Kirschner-wire fixation in distal radius fractures. Tech Hand Up Extrem Surg.

[13] Iacobellis C, Biz C (2014). Plating in diaphyseal fractures of the forearm. Acta bio-medica.

[14] Vasara H, Aspinen S, Kosola J, Sartanen J, Naalisvaara T, Myllykoski J, Stenroos A (2023). Adverse events after surgical treatment of adult diaphyseal forearm fractures: a retrospective analysis of 470 Patients. JB JS Open Access.

[15] Nappo KE, Hoyt BW, Balazs GC, Nanos GP, Ipsen DF, Tintle SM, Polfer EM (2019). Union rates and reported range of motion are acceptable after open forearm fractures in military combatants. Clin Orthop Relat Res.

[16] Mani GV, Hui PW, Cheng JC (1993). Translation of the radius as a predictor of outcome in distal radial fractures of children. J Bone Joint Surg.

[17] van Egmond PW, Schipper IB, van Luijt PA (2012). Displaced distal forearm fractures in children with an indication for reduction under general anesthesia should be percutaneously fixated. European J Orthop Surg Traumatol.

[18] Sengab A, Krijnen P, Schipper IB (2020). Risk factors for fracture redisplacement after reduction and cast immobilization of displaced distal radius fractures in children: a meta-analysis. European J Trauma Emerg Surg.

[19] Sengab A, Krijnen P, Schipper IB (2020). Risk factors for fracture redisplacement after reduction and cast immobilization of displaced distal radius fractures in children: a meta-analysis. European J Trauma Emerg Surg.

[20] Kong L, Lu J, Zhou Y, Tian D, Zhang B (2020). Incidence and risk factors for redisplacement after closed reduction and instant rigid cast immobilization for paediatric distal radius fractures: a case control study. J Orthop Surg Res.

[21] Mostafa MF, El-Adl G, Enan A (2009). Percutaneous Kirschner-wire fixation for displaced distal forearm fractures in children. Acta Orthop Belg.

[22] Khandekar S, Tolessa E, Jones S (2016). Displaced distal end radius fractures in children treated with Kirschner wires - a systematic review. Acta Orthop Belg.

[23] Ramoutar DN, Shivji FS, Rodrigues JN, Hunter JB (2015). The outcomes of displaced paediatric distal radius fractures treated with percutaneous Kirschner wire fixation: a review of 248 cases. European J Orthop Surg Traumatol.

[24] Miller BS, Taylor B, Widmann RF, Bae DS, Snyder BD, Waters PM (2005). Cast immobilization versus percutaneous pin fixation of displaced distal radius fractures in children: a prospective, randomized study. J Pediatr Orthop.

[25] Luscombe KL, Chaudhry S, Dwyer JS, Shanmugam C, Maffulli N (2010). Selective Kirschner wiring for displaced distal radial fractures in children. Acta Orthop Traumatol Turc.

[26] Pannu GS, Herman M (2015). Distal radius-ulna fractures in children. Orthop Clin North Am.

[27] Varga M, Józsa G, Fadgyas B, Kassai T, Renner A (2017). Short, double elastic nailing of severely displaced distal pediatric radial fractures: A new method for stable fixation. Medicine.

[28] Passiatore M, De Vitis R, Perna A, D'Orio M, Cilli V, Taccardo G (2020). Extraphyseal distal radius fracture in children: is the cast always needed? A retrospective analysis comparing Epibloc system and K-wire pinning. European J Orthop Surg Traumatol.

[29] De Vitis R, D'Orio M, Passiatore M, Perna A, Cilli V, Taccardo G (2022). Elastic stable intramedullary fixation using epibloc versus crossed Kirschner wires fixation for distal forearm fractures in children: a retrospective analysis. African J Paediatr Surg.

[30] van Egmond JC, Selles CA, Cleffken BI, Roukema GR, van der Vlies KH, Schep NWL (2019). Plate fixation for unstable displaced distal radius fractures in children. J Wrist Surg.

[31] Greig D, Silva M (2021). Management of distal radius fractures in adolescent patients. J Pediatr Orthop.

[32] - Waters PM, Skaggs DL, Flynn JM. (2019). Rockwood and Wilkins fractures in children. Lippincott Williams & Wilkins, Chapter 9, page 453

[33] Crosby BT, Behbahani A, Olujohungbe O, Cottam B, Perry D (2020). Developing a core outcome set for paediatric wrist fractures: a systematic review of prior outcomes. Bone Joint Open.

[34] Sallam AA, Briffa N, Mahmoud SS, Imam MA (2020). Normal wrist development in children and adolescents: a geometrical observational analysis based on plain radiographs. J Pediatr Orthop.

